# Accurate Detection of the Four Most Prevalent Carbapenemases in *E. coli* and *K. pneumoniae* by High-Resolution Mass Spectrometry

**DOI:** 10.3389/fmicb.2019.02760

**Published:** 2019-11-26

**Authors:** Dimard E. Foudraine, Lennard J. M. Dekker, Nikolaos Strepis, Michiel L. Bexkens, Corné H. W. Klaassen, Theo M. Luider, Wil H. F. Goessens

**Affiliations:** ^1^Department of Medical Microbiology and Infectious Diseases, Erasmus MC University Medical Center Rotterdam, Rotterdam, Netherlands; ^2^Department of Neurology, Neuro-Oncology Laboratory/Clinical and Cancer Proteomics, Erasmus MC University Medical Center Rotterdam, Rotterdam, Netherlands

**Keywords:** carbapenemases, *Eschericha coli*, *Klebsiella pneumoniae*, mass spectrometry – LC-MS/MS, parallel reaction monitoring, antimicrobial resistance

## Abstract

**Background:**

At present, phenotypic growth inhibition techniques are used in routine diagnostic microbiology to determine antimicrobial resistance of bacteria. Molecular techniques such as PCR are often used for confirmation but are indirect as they detect particular resistance genes. A direct technique would be able to detect the proteins of the resistance mechanism itself. In the present study targeted high resolution mass spectrometry assay was developed for the simultaneous detection of KPC, OXA-48-like, NDM, and VIM carbapenemases.

**Methods:**

Carbapenemase specific target peptides were defined by comparing available sequences in GenBank. Selected peptide sequences were validated using 62 *Klebsiella pneumoniae* and *Escherichia coli* isolates containing: 16 KPC, 21 OXA-48-like, 16 NDM, 13 VIM genes, and 21 carbapenemase negative isolates.

**Results:**

For each carbapenemase, two candidate peptides were validated. Method validation was performed in a blinded manner for all 83 isolates. All carbapenemases were detected. The majority was detected by both target peptides. All target peptides were 100% specific in the tested isolates and no peptide carry-over was detected.

**Conclusion:**

The applied targeted bottom-up mass spectrometry technique is able to accurately detect the four most prevalent carbapenemases in a single analysis.

## Introduction

The continuous increase of antimicrobial resistance in a diverse group of microorganisms is a major threat in the 21st century (WHO, 2014). Among several resistance mechanisms, carbapenemases are of particular concern. They not only hydrolyze carbapenem antibiotics, one of our last resort antibiotics, but the carbapenemases KPC, NDM, and VIM also hydrolyze cephalosporins with expanded-spectrum activity. Presence of additional resistance mechanisms results in multidrug resistance. Consequently, the treatment options for infections caused by carbapenemase-producing *Enterobacterales* (CPE) are limited (Stewart et al., 2018; Tacconelli et al., 2018).

To facilitate early adequate therapy for patients infected by CPE or other multidrug resistant bacteria, there is a need for new precise and rapid diagnostic methods (O’Neill, 2016). Shorter turnaround times for antimicrobial resistance determination leads to earlier appropriate antibiotic therapy and can contribute to a decrease in morbidity, mortality, length and cost of hospital stay, and can improve antibiotic stewardship (Kerremans et al., 2008; Perez et al., 2014; Pliakos et al., 2018).

In general, resistance determinations are performed by phenotypic growth inhibition assays which delay the time to result (van Belkum and Dunne, 2013; Zhou et al., 2018). Besides unfavorable turnaround time, phenotypic techniques are not optimal for detection of the different carbapenemase enzymes. This is especially the case for OXA-48-like enzymes with weak hydrolytic activity, often resulting in MIC’s below the “S”-breakpoint for carbapenems (Tamma and Simner, 2018). Furthermore, growth inhibition assays cannot classify the different carbapenemase enzymes. Classification of carbapenemases is crucial as new antibiotic combinations such as ceftazidime/avibactam can be effective against species producing KPC and OXA-48 but not against those producing NDM or VIM (Hawkey et al., 2018).

The presence of carbapenemases is generally confirmed by tests such as the Carbapenem Inactivation Method (CIM) or Carba NP (Meier and Hamprecht, 2019). These assays demonstrate carbapenemase activity but do not specify which carbapenemase is present.

PCR is also used for detection of carbapenemases. Although this technique has many benefits it does not provide information on the actual expression of the particular proteins (Traczewski et al., 2018; Schmidt et al., 2019). In contrast, lateral flow immunoassays do detect specific proteins rapidly and a recently marketed assay is able to detect the five main carbapenemases, i.e., KPC, OXA-48-like, NDM, VIM, and IMP with high sensitivity and specificity (Boutal et al., 2018).

New MS based methods could be an alternative to PCR and immunoassays (Charretier and Schrenzel, 2016; Oviano and Bou, 2019). In the last few years, several methods have been identified which detect carbapenemases indirectly by measuring hydrolysis of carbapenems using MALDI-TOF (Oviano and Bou, 2019). In contrast, direct and specific detection of prevalent carbapenemases such as KPC, NDM, OXA-48-like, and VIM by MALDI-TOF has not been demonstrated (Charretier and Schrenzel, 2016; Oviano and Bou, 2019). The MALDI-TOF which is currently used in clinical microbiology laboratories was designed to create a spectral fingerprint of abundant proteins. Therefore, its ability to directly detect low abundant proteins in complex samples is limited (Charretier and Schrenzel, 2016).

However, by combining high resolution mass spectrometry with liquid chromatography (LC) specific resistance mechanisms can be detected (Charretier and Schrenzel, 2016). Chromatographic separation and the use of a quadrupole to select for mass to charge ratios of target peptides can increase sensitivity, specificity and reproducibility of peptide detection (Charretier and Schrenzel, 2016; Banaei-Esfahani et al., 2017). Measurements can thereafter take place by selected reaction monitoring (SRM) in which selected fragments of selected peptides are measured; or parallel reaction monitoring (PRM) in which all peptide fragments of selected peptides are measured simultaneously, further increasing the selectivity of the assay (Guzel et al., 2018). High resolution targeted LC-MS/MS, either based on SRM or PRM could have potential for future resistance testing. By rapidly detecting specific peptides of many prevalent resistance mechanisms in a single assay, resistance to several representative antibiotics could be predicted for frequently isolated pathogens. So far, several studies have demonstrated direct detection of different resistance mechanisms using these targeted approaches (Charretier et al., 2015; Charretier and Schrenzel, 2016; Hassing et al., 2016). Furthermore, detection by high-resolution MS of each carbapenemase separately i.e., KPC (Wang et al., 2017), NDM (Cecchini et al., 2018), and OXA-48-like (Strich et al., 2019) has been demonstrated in different studies. However, in the present proof of concept study the aim was to apply LC-MS/MS for simultaneous detection of the prevalent key carbapenemases KPC, OXA-48-like, NDM, and VIM in cultures of *Escherichia coli* and *Klebsiella pneumoniae*.

## Materials and Methods

### Bacterial Isolates and PCR

In the present study, 83 strains were analyzed. Ethical approval was not required, as only stored bacterial isolates were used. The selected bacteria were: 63 *K. pneumoniae* consisting of 16 *K. pneumoniae* carbapenemase (KPC) positives, 17 oxacillinase-48-like (OXA-48-like) positives, 11 New Delhi metallo-β-lactamase (NDM) positives, 13 Verona integron-encoded metallo-β-lactamase (VIM) positives, and 10 carbapenem susceptible isolates; 20 *E. coli* consisting of 4 OXA-48-like positives, 5 NDM positives and 11 carbapenem-susceptible isolates. Of these, 2 KPC positive, 1 OXA-48-like positive, 2 NDM positive, 2 VIM positive, and 5 carbapenemase negative isolates were initially used for method development. Isolates were either obtained from the Erasmus MC collection or from the National Institute for Public Health and the Environment. VIM-positive *K. pneumoniae* isolates were obtained from the National School of Public Health, Athens, Greece. All strains were previously identified in our laboratory using the MALDI biotyper (Bruker, Billerica, MA, United States). Presence of KPC, OXA-48-like, NDM, and VIM enzymes was confirmed using in-house real-time PCR. The primers and probes that were used for PCR are listed in Supplementary Table 1.

### Peptide Selection

A BLASTn search was performed on the non-redundant GenBank database to find all available variants of *bla*_*KPC*_, *bla*_*NDM*_, *bla*_*VIM*_, and *bla*_*OXA–*__48__–like_. This included all curated subtypes (GenBank accession number PRJNA313047) as well as additional unique non-curated variants that were assigned a subtype and unannotated variants. Only full length or near full length hits were included. DNA sequences were translated into proteins and these were aligned using ClustalX (Larkin et al., 2007). Proteins were digested *in silico* with trypsin to identify tryptic candidate peptides for targeted MS. Peptides of 6–20 amino acids were selected based on maximum coverage of subtypes. Specificity of peptides in *Enterobacterales* was assessed using BLASTp. To select a suitable internal control peptide, 900 *K. pneumoniae* and *E. coli* reference genomes were randomly selected from GenBank. Sequences were subsequently annotated with Prokka v1.13 and translated into proteins (Seemann, 2014). Ribosomal protein S1 was present in all genomes. From this protein, a conserved peptide of suitable length was selected which was identical in all strains.

### Culture Protocol

Isolates stored at −80°C were subcultured on Trypticase^TM^ Soy Agar II plates with 5% sheep blood (Becton Dickinson, Franklin Lakes, NJ, United States) and incubated overnight at 37°C. Subsequently, one inoculation loop with bacteria was inoculated in Mueller Hinton II broth and cultured overnight at 37°C and 150 rpm. Resulting broth was diluted with Mueller Hinton II broth until absorbance of 0.98–1.02 at 600 nm wavelength was reached. Subsequently, 1 ml was centrifuged for 5 min at 21,000 × *g*. The resulting pellet was washed once with 500 μl phosphate-buffered saline and stored at −20°C.

### Lysis and Digestion Protocol

**FIGURE 1:**
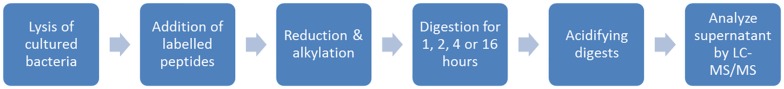
Schematic overview of the LC-MS/MS pre-treatment used in this study.

A schematic overview of the lysis and digestion protocol is shown in Figure 1. Bacterial pellets were dissolved in 100 μl lysis solution containing 5% sodium deoxycholate and 7.5 mM dithiothreitol in water. Ten microliter of stable isotope-labeled (SIL) peptide solution was added containing 5 pmol labeled OXA-48-like and NDM peptides, 1 pmol KPC peptides and 0.5 pmol VIM peptides. SIL variant peptides were obtained of selected target peptides via Pepscan, Lelystad, Netherlands. Concentrations of SIL peptides were optimized in a pilot experiment to obtain ratios to target peptides close to one. Samples were sonicated for 5 min (Branson 2510 Ultrasonic Cleaner, Branson Ultrasonics, Danbury, United States) and incubated for 10 min at 80°C, 450 rpm in a heater-shaker (Eppendorf ThermoMixer^®^ C, Eppendorf, Hamburg, Germany). One and a half microliter of 500 mM dithiothreitol was added and samples were incubated for 20 min at 60°C and 450 rpm. Subsequently, 7 μl 375 mM iodoacetamide was added followed by 30 min of incubation in the dark at room temperature. Samples were diluted 10-fold using MS grade water, 1 μl trypsin (1 μg/μl) (Worthington, NJ, United States) was added followed by 25 μl Tris–HCl buffer (pH 8). In general, samples were incubated overnight, however, different trypsin incubation periods of 1, 2, and 4 h were tested in separate experiments (37°C, 450 rpm). After trypsinization, 20 μl 5% trifluoroacetic acid was added followed by 30 min of incubation at 37°C and 450 rpm. Digests were centrifuged for 60 min at 4°C, 21,000 × *g* and 500 μl of supernatant was stored at 5°C awaiting LC-MS/MS.

### LC-MS/MS

Digests were loaded onto Evotips (Evosep, Odense, Denmark) according to the manufacturer’s instructions (Bache et al., 2018). Evotips were soaked in isopropanol and sequentially washed with 20 μl acetonitrile with 0.1% formic acid, equilibrated with 20 μl water with 0.1% formic acid (solvent A), loaded with 20 μl sample, washed with 20 μl solvent A and loaded with 100 μl solvent A. Tips were centrifuged for 60 s at 700 × *g* between each loading step. The Evotips were submerged in 0.1% formic acid on a 96-wells plate during sample loading to prevent drying. After the last step, tips were centrifuged for 20 s at 700 × *g*. LC was performed by the Evosep One (Evosep, Odense, Denmark) using the manufactures separation method of 11.5 min (100 samples/day) (Bache et al., 2018). The Evosep One was coupled to an Orbitrap mass spectrometer (Q Exactive HF Hybrid Quadrupole-Orbitrap, Thermo Fisher Scientific, Bremen, Germany). The Q Exactive HF system was operated in PRM mode using the following parameters: a quadrupole isolation window of 0.6 m/z units, an automatic gain control target value of 1 × 10^6^ ions, a maximum fill time of 150 ms and a resolving power of 60,000 at 200 m/z. A normalized collision energy of 27% was used for all peptides. A retention time window of 2 min was used for each peptide.

### Data Analysis

Raw data was loaded and analyzed using Skyline (Skyline 3.7 or later, MacCoss Lab Software, University of Washington, United States). Spectral peak areas of target peptides were calculated by summing up fragment peak areas. For each target the ratio between the peak area of the endogenous peptide and of the SIL peptide was calculated. A peptide was considered present when the following criteria were met: Retention time similar to that of the SIL peptide, presence of all fragments, ratio dot products (rdotp) >0.95 and mass error <5 ppm. The rdotp is a measure of similarity between the fragment ratio of the endogenous and SIL peptide (Strich et al., 2019). Results were compared with PCR for all samples. Sensitivity, specificity and their respective 95% CI were calculated using VassarStats: Website for Statistical Computation^1^. Coefficients of variation (CV) were calculated using Microsoft Excel.

## Results

### Detection of Candidate Peptides

**FIGURE 2:**
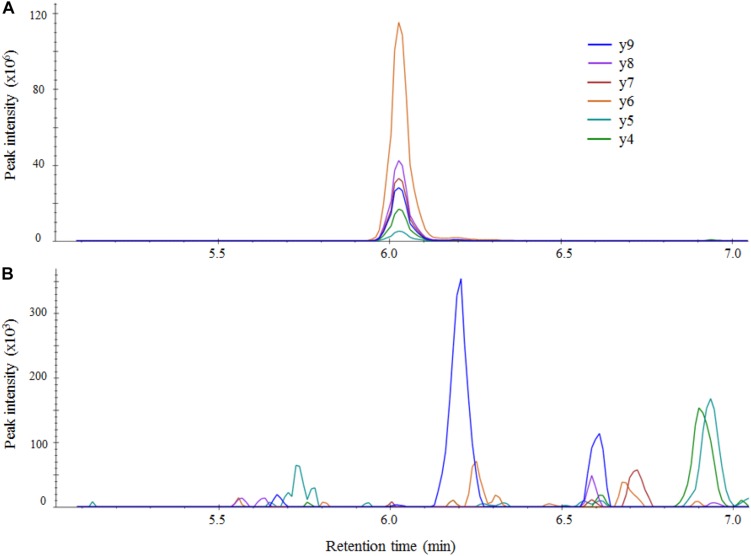
LC-MS/MS chromatograms of the OXA-48-like peptide ANQAFLPASTFK in Skyline for an OXA-48-like positive sample **(A)** and an OXA-48-like negative sample **(B)**. The negative sample was measured right after the strong positive sample and no carry-over of the target peptide was detected. Intensity of fragments in unadjusted units (multiplied by 10^6^ for the positive sample and 10^3^ for negative sample) is plotted against retention time in minutes.

From the protein analysis of the four target genes i.e., KPC, OXA-48-like, NDM, and VIM, several peptides were selected *in silico* as target peptides to be used as markers for presence of the aforementioned carbapenemases. All candidate peptides are displayed in Supplementary Table 2. Suitability of candidate peptides was tested on a selected panel of 12 isolates containing all four carbapenemase genes. The aim was to select two suitable peptides for each carbapenemase. For the detection of the KPC-carbapenemase, four candidate peptides were tested. The peptides GFLAAAVLAR and APIVLAVYTR were detected in both KPC-positive samples, however, peak areas were extremely variable making them less suitable as reliable markers. Peptides SIGDTTFR and FPLCSSFK were also detected in both samples and peak areas were similar. Therefore, these were selected for further validation. In the detection of the OXA-48-like and NDM carbapenemases the first two peptides were tested, all four showed good results so no other candidates were tested. In the detection of the VIM-carbapenemase, candidate peptides DGDELLLIDTAWGAK, VGGVDVLR, and NTAALLAEIEK were tested. As the latter two showed higher peak areas they were selected for further validation. The peptide selected as internal control HEAWITLEK was detected in all 12 samples. The final peptide panel used for validation consisted of the eight selected carbapenemase peptides and the internal control peptide (Table 1). To assess possible sample carry-over, several negative samples were analyzed preceded by known positive samples. No carry-over was detected for any of the selected peptides (e.g., Figure 2).

**TABLE 1:** Selected target peptides for detection of the carbapenemases KPC, OXA-48-like, NDM, VIM, and 30S ribosomal protein (internal control).

**Carbapenemase**	**Peptide**	**Variants included**	**Charge**	**Mass (m/z)**	**Retention window (min)**	**Fragment ions analyzed**
KPC	SIGDTTFR	*bla*KPC-1 to 19, 21, 22, 24 to 26, 28 and 1 unannotated subtype	2+	448.72725	2.58–4.58	y6, y5, y4, y3
	FPLCSSFK	as above	2+	493.24422	4.62–6.62	y7^∗^, y6, y5, y4

OXA-48-like	ANQAFLPASTFK	*bla*OXA-48, 162, 163, 181, 199, 204, 232, 244, 245, 247, 370 and 3 unannotated subtypes	2+	647.84314	5.08–7.08	y9, y8, y7, y6, y5, y4
	SWNAHFTEHK	*bla*OXA-48, 162, 163, 181, 204, 232, 244, 245, 247, 370	3+	419.53175	2.03–4.03	y8, y7, y6, y5, y4, y3

NDM	FGDLVFR	*bla*NDM-1 to 16 and 1 unannotated subtype	2+	427.23197	5.49–7.49	y6, y5, y4, y3
	QEINLPVALAVVTHAHQDK	as above	4+	521.53782	6.49–8.49	y8, y7, y6, y5, y4, b5

VIM	VGGVDVLR	*bla*VIM-1 to 12, 14 to 20, 23 to 46, 49 to 51 and 11 unannotated subtypes	2+	407.74270	3.06–6.06	y7, y6, y5, y4, y3
	NTAALLAEIEK	*bla*VIM-1 to 6, 8 to 12, 14 to 20, 23 to 46, 49 to 51 and 11 unannotated subtypes	2+	586.82970	6.06–8.06	y9, y7, y5, y4

	HEAWITLEK (internal control – 30S ribosomal reference protein)		2+	563.79821	3.95–5.95	y8, y7, y6, y5, y4, y3 b3, b4, b5, b6, b7, b8

*All fragment ions had a charge of +1 except for y7 of FPLCSSFK (^∗^) which had a charge of +2.*

### Specificity Assessment Using BLASTp

Specificity of the selected KPC, OXA-48-like, NDM, and VIM peptides was assessed by performing BLASTp searches for the peptides in *Enterobacterales*. Results showed the KPC peptide SIGDTTFR was also present in the class A carbapenemase SED found in *Citrobacter* isolates. The other peptide selected for KPC detection, i.e., FPLCSSFK was also present in the low prevalent class A carbapenemases FRI, SME, IMI, and NMC found in various *Enterobacterales*. None of the peptides selected for detection of OXA-48-like, NDM, and VIM enzymes were present in any other protein.

### Reproducibility Assessment

To assess the robustness of the method, reproducibility was tested using two strains positive for both OXA-48-like and NDM carbapenemases and two strains positive for both KPC and VIM. Five ml cultured broth of each strain was distributed over five aliquots and processed separately. CV’s of endogenous to SIL peptide ratios were determined for all peptides (Table 2). Mean CV’s were 15.2% for SIGDTTFR (KPC), 22.5% for FPLCSSFK (KPC), 9.4% for ANQAFLPASTFK (OXA-48-like), 10.7% for SWNAHFTEHK (OXA-48-like), 54.2% for FGDLVFR (NDM), 9.4% for VGGVDVLR (VIM), and 13.2% for NTAALLAEIEK (VIM). The CV of QEINLPVALAVVTHAHQDK was not determined as the peptide was false negative in both NDM-positive samples.

**TABLE 2:** Intensity ratios and coefficients of variation (CV) of the eight target peptides using two strains positive for both OXA-48-like and NDM and two strains positive for both KPC and VIM.

**Carbapenemase**	**Target peptide**	**Range of intensity ratios first sample**	**CV first sample (%)**	**Range of intensity ratios second sample**	**CV second sample (%)**
KPC	SIGDTTFR	0.60–0.79	11.5	0.50–0.78	18.9
	FPLCSSFK	0.67–1.40	28.5	0.62–0.95	16.5
OXA-48-like	ANQAFLPASTFK	0.98–1.18	7.8	1.14–1.50	11.0
	SWNAHFTEHK	5.95–7.29	7.4	6.89–9.91	14.0
NDM	FGDLVFR	0.83–2.23	51.1	0.98–4.59	57.3
	QEINLPVALAVVTHAHQDK	^∗^	^∗^	^∗^	^∗^
VIM	VGGVDVLR	14.35–18.35	9.5	14.70–18.76	9.3
	NTAALLAEIEK	0.43–0.63	14.4	0.38–0.48	12.0

*Each strain was processed in fivefold. Ratios were calculated by dividing peak areas of endogenous peptides to stable isotope-labeled peptides. CV’s were calculated of these ratios. ^∗^QEINLPVALAVVTHAHQDK was not detected in both samples.*

### Method Validation

**FIGURE 3:**
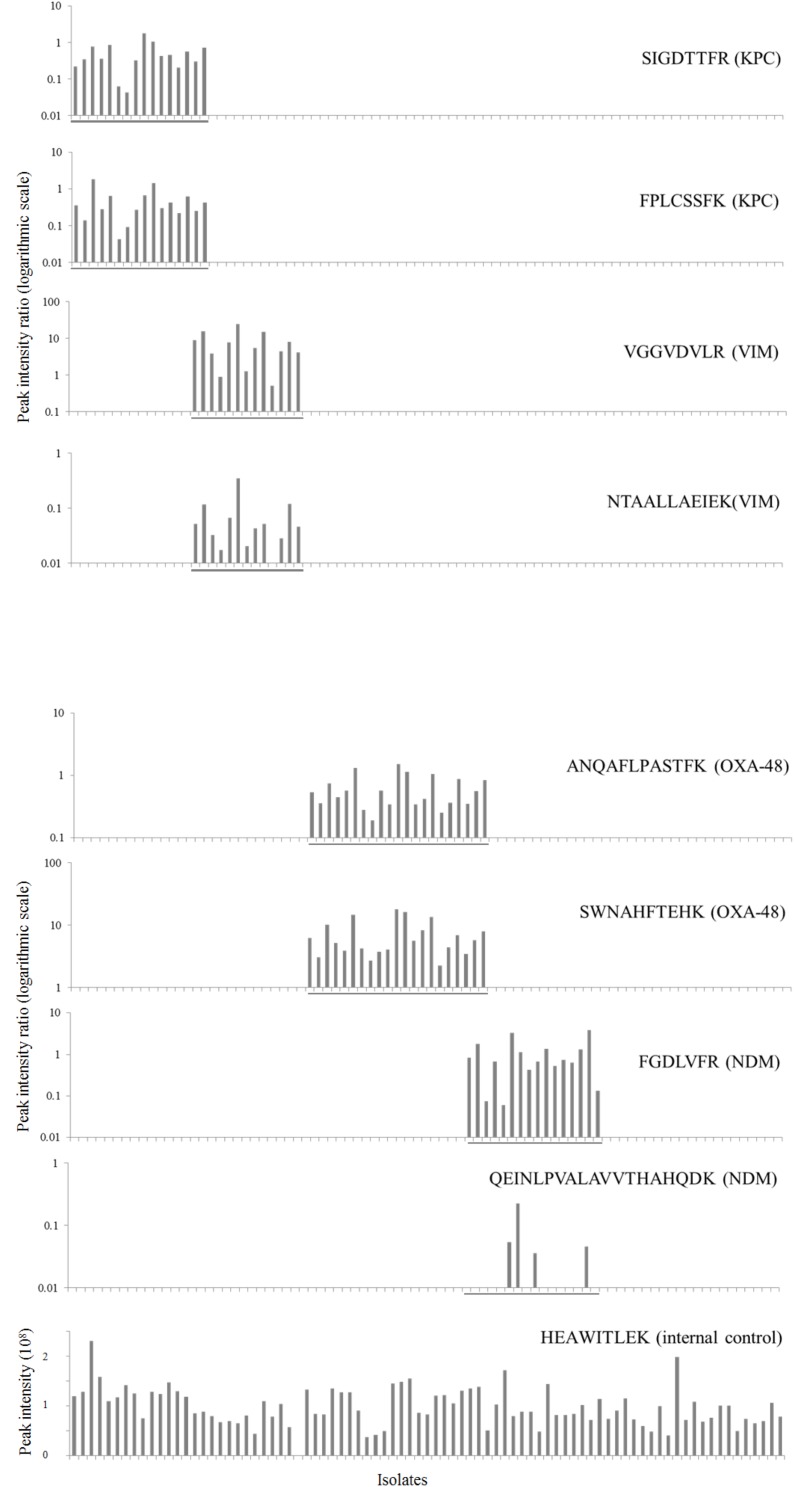
Validation of the LC-MS/MS technique in 83 isolates. Intensity ratios of selected KPC, VIM, OXA-48-like and NDM endogenous target peptides to spiked stable isotope-labeled reference peptides (logarithmic scale) are plotted for each isolate. PCR positive isolates are grouped together in the figure and marked by a line for each respective carbapenemase. The bottom graph shows intensity of the ribosomal protein which was selected as internal control in unadjusted units (10^8^).

After *in silico* and experimental selection of the eight target peptides and reproducibility assessment, the method was validated using all 83 previously characterized isolates in a blinded manner. All carbapenemase enzymes were detected by the assay (Table 3). However, not all selected peptides were 100% sensitive. The VIM-peptide NTAALLAEIEK did miss one out of 13 VIM positive *K. pneumoniae* isolates (sensitivity 92%, 95% CI, 62–100%). Furthermore, the NDM-peptide QEINLPVALAVVTHAHQDK (NDM) was detected in only 4 out of 16 isolates resulting in a sensitivity of 25% (95% CI, 8–53%). All other peptides were 100% sensitive. All peptides were 100% specific and no carry-over was detected. Interference of background signal did not lead to false positive results as there were no interfering peaks which met the detection criteria. The internal control peptide of the 30 S ribosomal protein S1 was present in all samples except for one VIM positive *K. pneumoniae* isolate. To optimize the accuracy of peptide detection, SIL variants of each target peptide were added during the extraction procedure. Ratios of endogenous to SIL peptides are depicted in Figure 3 for all 83 isolates. It was observed that the ratios for individual peptides covered a dynamic range of nearly three orders of magnitude. Even for the samples with the lowest abundance of target peptides, a clear signal which passed all detection criteria was still observed.

**TABLE 3:** Sensitivity and specificity of LC-MS/MS compared to PCR in 83 strains.

**Carbapenemase**	**Target peptide**	**Sensitivity %, CI %, (n MS positive/n PCR positive)**	**Specificity %, CI %, (n MS negative/n PCR negative)**
KPC	SIGDTTFR	100, 76–100 (16/16)	100, 93–100 (67/67)
	FPLCSSFK	100, 76–100 (16/16)	100, 93–100 (67/67)
	One or both KPC peptides positive	100 (16/16)	100 (67/67)
OXA-48-like	ANQAFLPASTFK	100, 81–100 (21/21)	100, 93–100 (62/62)
	SWNAHFTEHK	100, 81–100 (21/21)	100, 93–100 (62/62)
	One or both OXA-48-like peptides positive	100 (21/21)	100 (62/62)
NDM	FGDLVFR	100, 76–100 (16/16)	100, 93–100 (67/67)
	QEINLPVALAVVTHAHQDK	25, 8–53 (4/16)	100, 93–100 (67/67)
	One or both NDM peptides positive	100 (16/16)	100 (67/67)
VIM	VGGVDVLR	100, 72–100 (13/13)	100, 94–100 (70/70)
	NTAALLAEIEK	92, 62–100 (12/13)	100, 94–100 (70/70)
	One or both VIM peptides positive	100 (13/13)	100 (13/13)

### Assessment of Protocol With Shorter Digestion Times

For the validation set, samples were incubated overnight for trypsin digestion. To assess whether shorter trypsin digestion times would compromise sensitivity, two strains positive for both OXA-48-like and NDM carbapenemases and two strains positive for both KPC and VIM were cultured in broth and four ml of each strain was distributed over four aliquots. Each was incubated with trypsin, either for 1, 2, 4 or 16 h. NTAALLAEIEK (VIM), VGGVDVLR (VIM), SIGDTTFR (KPC), FGDLVFR (NDM), SWNAHFTEHK (OXA-48-like), and ANQAFLPASTFK (OXA-48-like) were all positive according to our detection criteria in the four samples digested for 1 h, albeit with lower ratios to reference peptides. Optimal detection of FPLCSSFK (KPC) required at least 2 h before all detection criteria were met and QEINLPVALAVVTHAHQDK (NDM) even required overnight digestion. These results demonstrate that shorter trypsin incubation times are feasible without compromising the sensitivity of the assay provided to include only the best performing peptides.

## Discussion

In an era of increasing antimicrobial resistance new technologies are needed to facilitate early optimal antibiotic therapy and timely infection prevention measures (van Belkum and Dunne, 2013; Perez et al., 2014; WHO, 2014; The Review on Antimicrobial Resistance, 2016; Tacconelli et al., 2018). PCR and WGS are frequently used as confirmation techniques or even cited as techniques replacing conventional AST. However, PCR is limited in multiplexing capacity and WGS is at present still expensive and not yet suited to be a routine method for the most frequently isolated pathogens (Ellington et al., 2017). Lateral flow immunoassays are an alternative to PCR and can be used for rapid detection of some resistance mechanisms (Boutal et al., 2018; Volland et al., 2019).

A technique able to detect most prevalent resistance mechanisms simultaneously, accurately and rapidly would be beneficial to prevent further spread of AMR and initiate optimal therapy in an early stage of infection. Although targeted LC-MS/MS is not routinely used for detection of resistance mechanisms, it does offer some advantages. It is less hampered by multiplexing issues than PCR and immunoassays. Furthermore, results can be obtained rapidly when sample pre-treatment time and organism enrichment can be by-passed or shortened (Charretier et al., 2015; Wang et al., 2017; Saleh et al., 2019; Strich et al., 2019).

To further explore the possibilities of high resolution mass spectrometry in detecting resistance mechanisms, a mass spectrometry workflow was developed and tested for detection of the most prevalent carbapenemases in a single analysis. As proof of principle, 83 isolates consisting of *E. coli* and *K. pneumoniae* were tested for the presence of KPC, OXA-48-like, NDM and VIM enzymes. All present carbapenemases were detected, either by detecting one of the two selected target peptides or both, which was the case for the majority of isolates.

In contrast to other studies that demonstrated direct detection of KPC, OXA-48-like and NDM carbapenemases using targeted LC-MS/MS we broadened the detectable spectrum by VIM (Wang et al., 2017; Cecchini et al., 2018; Strich et al., 2019). Furthermore, it was demonstrated that it is technically feasible to measure four carbapenemases in a single assay with 100% sensitivity and specificity. This was in part possible due to the use of PRM instead of SRM. The first shown in a recent study, to have a lower limit of detection and better overall performance in complex samples (Guzel et al., 2018).

In the current assay, 9 endogenous and 8 SIL peptides were measured in a single analysis. It is however, technically feasible to detect many more different peptides at the same time. In a study of Charretier et al. (2015), 109 endogenous *Staphylococcus aureus* and 49 synthesized peptides were measured in a single experiment by SRM although this did extend the LC gradient and MS analysis to 34 min for each sample.

In addition to carbapenemases, other resistance mechanisms of *Enterobacterales* have been detected by LC-MS such as the ESBL CTX-M by Fleurbaaij et al. (2017). But also the detection of different amino-acid substitutions in the GyrA protein resulting in quinolone resistance of *Salmonella enterica* isolates was demonstrated in a paper of Hassing et al. (2016). These studies indicate that LC-MS is universally applicable for protein detection of different resistance mechanisms. By expanding the current detection of carbapenemases by ESBL’s, mutated gyrases, aminoglycoside modifying enzymes and even absence of porins, a single MS assay has the potential to detect many different resistance mechanisms simultaneously. Hypothetically, even the combined presence of two resistance mechanisms affecting the same antibiotic could be detected. For example, a lack of porins combined with beta-lactamase production leading to reduced susceptibility to carbapenems (Goessens et al., 2013).

To achieve a high sensitivity in detecting different resistance mechanisms, it is important to take different subtypes into account. In the current selection process, all subtypes of each carbapenemase gene were taken into consideration. This resulted in a high coverage of each carbapenemase. To illustrate: all new CPE isolates recovered from patients in the Netherlands are characterized by the National Institute for Public Health and Environment. In 2017, 235 new CPE isolates were submitted of which 220 (93.6%) had a known subtype (Mouton and de Greeff, 2018). All of these subtypes were covered by our selected peptides except for one OXA-23 (which is obvious as it is not part of the OXA-48 family) (Mairi et al., 2018).

In addition to being sensitive, peptides should also be specific. In this study specificity was assessed *in vitro* and by BLASTp searches for the target peptides. All carbapenemase peptides were specific for their respective resistance mechanisms in *Enterobacterales* except for the two KPC peptides. Although FPLCSSFK aligned with multiple other class A carbapenemases, SIGDTTFR was only present in KPC and the class A carbapenemase SED produced by *Citrobacter* species. Furthermore, it should be noted that although the peptides selected for “OXA-48-like” are specific for the OXA-48 family, the enzymes OXA-163, OXA-247, and OXA-405 within this family will be detected but do not hydrolyze carbapenems (Strich et al., 2019).

In addition to the specific carbapenemase peptides, a 30 S ribosomal protein was added to the assay as an internal control for which 82/83 strains tested positive. Because a negative result was remarkable for such an abundant protein, WGS was performed on the internal control negative isolate. This demonstrated a mutation in the target gene which led to the peptide sequence HEAWISLEK instead of HEAWITLEK, explaining why this strain tested negative.

The present results are encouraging, especially when reducing the turnaround time, making the technique more suitable for routine diagnostics. The pre-treatment protocol currently used was not yet optimized to be as short and simple as possible. However, in a pilot it was demonstrated that all four carbapenemases can be detected within a few hours using shorter digestion times. In a study by Wang et al. (2017), total time from culture to results was reduced to 90 min for detection of KPC using a relatively simple pre-treatment protocol. The currently used pre-treatment protocol and the protocol used by Wang et al. (2017) are both relatively easy to automate which would further decrease turnaround time and increase applicability (Sabbagh et al., 2016).

Besides turnaround time, it is also important to decrease hands-on time per sample, as most laboratories analyze high numbers of isolates making automation of this technique a requisite. In the present study, the Evosep One was used as an integrated liquid chromatograph and auto-sampler which reduced turnaround time. The system had two other benefits. First, no carryover was detected making blank runs redundant. Second, high sample throughput was demonstrated using an 11.5 min elution gradient, potentially analyzing 100 samples a day.

Although the present study was “proof of concept,” the results show the potential of targeted high resolution MS in detecting different resistance mechanisms. If the target panel would be expanded by peptides specific for aminoglycoside modifying enzymes, gyrase mutations, penicillinases (such as SHV and TEM), and ESBLs (such as CTX-M), rapid screening of for instance positive blood cultures could be beneficial, provided that high-resolution MS is available in a routine setting. By using the bacteria present in positive blood cultures, subsequent processing and analysis by targeted MS could result in early information on the presence of these resistance mechanisms. This could in turn facilitate an earlier switch to the most appropriate therapy. However, before considering application in a routine setting further validation using more strains with different resistance mechanisms is warranted.

In summary, a bottom up LC-MS/MS method was developed which could accurately detect KPC, OXA-48-like, NDM and VIM carbapenemases and which has the potential to rapidly detect many other resistance mechanisms in a single assay as well.

## Data Availability Statement

All datasets generated for this study are included in the article/Supplementary Material.

## Author Contributions

WG, TL, LD, CK, and DF designed the study. CK and NS selected the peptide markers *in silico*. DF and LD performed the experiments and analyzed the data. DF, WG, and LD wrote the manuscript. All authors edited and approved the manuscript.

## Conflict of Interest

The authors declare that the research was conducted in the absence of any commercial or financial relationships that could be construed as a potential conflict of interest. The Erasmus MC is patent holder of “mass spectrometric determination of drug resistance” (PCT/NL2013/050255) which is licensed via Da Vinci Laboratory Solutions (Rotterdam, Netherlands).

## Footnotes

1 http://vassarstats.net
