# Supplementary material for: Accurate Detection of the Four Most Prevalent Carbapenemases in E. coli and K. pneumoniae by High-Resolution Mass Spectrometry
Source: Front Microbiol. 2019 Nov 26;10:2760. doi: 10.3389/fmicb.2019.02760 (PMC6901907; doi:10.3389/fmicb.2019.02760)
Supplement: Supplementary file 1 [file Data_Sheet_1.docx]

**Supplementary table 1.** PCR primers and probes used to detect the carbapenemases KPC, OXA-48, NDM and VIM.

| KPC | Forward primer  Reverse primer  Probe  Reference | 5’-TGCAGAGCCCAGTGTCAGTTT-3’  5’-CGCTCTATCGGCGATACCA-3’  5’-TTCCGTCACGGCGCGCG-3’  * |
| --- | --- | --- |
| OXA-48 | Forward primer  Reverse primer  Probe  Reference | 5’-GCGAACCAAGCATTTTTACC-3’  5’-ATCCTTAACCACGCCCAAAT-3’  5’-CCCAATAGCTTGATCGCCCTCG-3’  This study |
| NDM | Forward primer  Reverse primer  Probe  Reference | 5’-CATTAGCCGCTGCATTGATG-3’  5’-GTCGCCAGTTTCCATTTGCT-3’  5’-CATGCCCGGTGAAATCCGCC-3’  * |
| VIM | Forward primer  Reverse primer 1  Reverse primer 2  Probe  Reference | 5’-GCAAATTGGACTTCCYGTAA-3’  5’-GACGGTGATGCGTACGTTG-3’  5’-CCCTAAGGGCATCAACTCC-3’  5’-TTTCATGACGACCGCGTCGG-3’  This study |

* A. van der Zee, L. Roorda, G. Bosman, A.C. Fluit, M. Hermans, P.H. Smits, A.G. van der Zanden, R. Te Witt, L.E. Bruijnesteijn van Coppenraet, J. Cohen Stuart, and J.M. Ossewaarde, Multi-centre evaluation of real-time multiplex PCR for detection of carbapenemase genes OXA-48, VIM, IMP, NDM and KPC. BMC Infect Dis 14 (2014) 27.

**Supplementary table 2.** Candidate peptides for detection of the carbapenemases KPC, OXA-48, NDM and VIM.

| Carbapenemase | Candidate peptides | Variants included |
| --- | --- | --- |
| KPC | GFLAAAVLAR  APIVLAVYTR  SIGDTTFR  FPLCSSFK  AVTESLQK  GNTTGNHR  AAVPADWAVGDK | blaKPC-1 to 19, 21, 22, 24 to 26, 28 and 1 unannotated subtype  as above  as above  as above  as above  as above  as above |
| OXA-48 | ANQAFLPASTFK  SWNAHFTEHK  SQGVVVLWNENK  QQGFTNNLK  QAMLTEANGDYIIR  IPNSLIALDLGVVK  DEHQVFK  YSVVPVYQEFAR | blaOXA-48, 162, 163, 181, 199, 204, 232, 244, 245, 247, 370 and 3 unannotated subtypes  blaOXA-48, 162, 163, 181, 204, 232, 244, 245, 247, 370  as above  blaOXA-48, 162, 163, 181, 199, 204, 232, 244, 245, 247, 370  as above  blaOXA-48, 162, 163, 181, 199, 204, 232, 244, 245, 247, 370 and 3 unannotated subtypes  as above  blaOXA-48, 162, 163, 181, 199, 204, 232, 244, 247, 370 and 3 unannotated subtypes |
| NDM | FGDLVFR  QEINLPVALAVVTHAHQDK  AFGAAFPK  ASMIVMSHSAPDSR  PTIGQQMETGDQR  AAITHTAT | blaNDM-1 to 16 and 1 unannotated subtype  as above  as above  as above  blaNDM-1 to 9, 11 to 16 and 1 unannotated subtype  blaNDM-1 to 15 and 1 unannotated subtype |
| VIM | DGDELLLIDTAWGAK  NTAALLAEIEK  VGGVDVLR  QIGLPVTR  AVSTHFHDDR | blaVIM-1 to 6, 8 to 12, 14 to 20, 23 to 46, 49 to 51 and 11 unannotated subtypes  as above  blaVIM-1 to 12, 14 to 20, 23 to 46, 49 to 51 and 11 unannotated subtypes  blaVIM-1 to 20, 23 to 47, 49 to 51 and 11 unannotated subtypes  blaVIM-1 to 6, 8 to 12, 14 to 20, 23 to 33, 35 to 46, 49 to 51 and 11 unannotated subtypes |
